# EBNA2 driven enhancer switching at the CIITA-DEXI locus suppresses HLA class II gene expression during EBV infection of B-lymphocytes

**DOI:** 10.1371/journal.ppat.1009834

**Published:** 2021-08-05

**Authors:** Chenhe Su, Fang Lu, Samantha S. Soldan, R. Jason Lamontagne, Hsin-Yao Tang, Giorgia Napoletani, Paul J. Farrell, Italo Tempera, Andrew V. Kossenkov, Paul M. Lieberman

**Affiliations:** 1 The Wistar Institute, Philadelphia, Pennsylvania, United States of America; 2 Imperial College, London, United Kingdom; University of Wisconsin-Madison, UNITED STATES

## Abstract

Viruses suppress immune recognition through diverse mechanisms. Epstein-Barr Virus (EBV) establishes latent infection in memory B-lymphocytes and B-cell malignancies where it impacts B-cell immune function. We show here that EBV primary infection of naïve B-cells results in a robust down-regulation of HLA genes. We found that the viral encoded transcriptional regulatory factor EBNA2 bound to multiple regulatory regions in the HLA locus. Conditional expression of EBNA2 correlated with the down regulation of HLA class II transcription. EBNA2 down-regulation of HLA transcription was found to be dependent on CIITA, the major transcriptional activator of HLA class II gene transcription. We identified a major EBNA2 binding site downstream of the CIITA gene and upstream of DEXI, a dexamethasone inducible gene that is oriented head-to-head with CIITA gene transcripts. CRISPR/Cas9 deletion of the EBNA2 site upstream of DEXI attenuated CIITA transcriptional repression. EBNA2 caused an increase in DEXI transcription and a graded change in histone modifications with activation mark H3K27ac near the DEXI locus, and a loss of activation marks at the CIITA locus. A prominent CTCF binding site between CIITA and DEXI enhancers was mutated and further diminished the effects of EBNA2 on CIITA. Analysis of HiC data indicate that DEXI and CIITA enhancers are situated in different chromosome topological associated domains (TADs). These findings suggest that EBNA2 down regulates HLA-II genes through the down regulation of CIITA, and that this down regulation is an indirect consequence of EBNA2 enhancer formation at a neighboring TAD. We propose that enhancer competition between these neighboring chromosome domains represents a novel mechanism for gene regulation demonstrated by EBNA2.

## Author summary

We describe a novel transcriptional mechanism through which EBV encoded EBNA2 down-regulates HLA class II gene expression. EBNA2 is known to be a potent transcriptional activator and strengthens enhancer formation at many sites in B-lymphocytes. We show that EBNA2 binds to an enhancer element at the DEXI gene that competes with CIITA gene promoter, leading to the down regulation of CIITA transcription. As CIITA is the master regulator of HLA class II gene expression, these findings reveal a novel, highly indirect mechanism for EBNA2 down-regulation of HLA II gene transcription. Down regulation of HLA class II gene expression by EBNA2 is functionally important for the attenuation of T-cell recognition of EBV infected cells, and likely to contribute to EBV persistence and lymphomagenesis *in vivo*.

## Introduction

Virus down-regulation of HLA is a classic mechanism of immune evasion [1]. Epstein-Barr Virus (EBV) is a human γ-herpesvirus that establishes long-term latent infection in memory B-cells, and is also associated with various malignancies, including Burkitt’s lymphoma, Hodgkin’s Lymphoma, and Non-Hodgkin’s Lymphomas, nasopharyngeal carcinoma and subtypes of gastric carcinoma [2–5]. EBV’s success in establishing latent infection is due, in part, to its capacity to evade immune cell recognition [6,7]. EBV has been shown to evade immune recognition through numerous mechanisms, including the inhibition of antigen presentation and down-regulation of HLA gene expression [8–10].

The human leukocyte antigen (HLA) locus contains clusters of genes involved in antigen presentation and immune signaling [11,12]. It is also among the most genetically polymorphous regions due to the high rates of evolutionary competition with infectious agents [13]. Polymorphisms in the HLA locus are most frequently associated with risk to infection and auto-immune diseases, including multiple sclerosis, systemic lupus erythematosus, and diabetes [14–16]. While all cells display self-antigens through class I HLA, professional antigen presenting cells, including B-lymphocytes, process foreign antigens through class II HLA for presentation to T-cells [11,12]. Many viruses evade host immune recognition by down regulating antigen presentation by components of the HLA system through various and diverse mechanisms [17–19].

EBV encodes several genes known to alter host immune function [6,20]. During productive infection BNLF2 inhibits HLA antigen processing by directly blocking the transporter protein TAP [21,22]. During lytic reactivation, BZLF1 inhibits transcription of HLA class II chaperone CD74 [23], and the master regulator of class II gene transcription CIITA [24,25]. During latency EBNA1 suppresses its own HLA presentation by interfering with peptide processing [26,27]. The latency membrane protein LMP2A that mimics BCR signaling correlates with down-regulation of HLA-class I genes and DNA hypermethylation of the HLA locus [28]. Latency membrane protein 1 (LMP1) required for CD40-like signaling and B-cell immortalization down-regulates HLA class I expression in B-lymphocyte [29], but may have the opposite effect in epithelial cells [30]. Thus, EBV can modulate HLA I and II gene expression through various mechanisms depending on the stage of viral life cycle and the cell or tumor type.

EBNA2 is a potent transcriptional regulator essential for EBV primary infection and immortalization of B-cells [31–33]. EBNA2 is known to rewire B-cell transcriptional control to promote transition from resting B-cell to highly proliferative germinal center blast [34]. EBNA2 does not bind directly to DNA, like EBNA1, but rather interacts with several cellular sequence specific transcription factors, including RBPJ, EBF1, and PU.1 [35,36]. EBNA2 also interacts with several transcriptional co-activators, including SNF5 and p300, and is thought to function primarily through the formation of new super-enhancers [34,37]. EBNA2 can also facilitate the formation of new chromosome binding sites for EBF1 and RBPJ to activate target genes [38].

Conversely, EBNA2 can also down-regulate some genes, such as BCL6 and TCL1 to restrict the germinal center phenotype [39]. The mechanisms through which EBNA2 down-regulates transcription of some genes are not well-understood.

Here, we show that EBV primary infection of B-lymphocytes results in a concerted down regulation of HLA II genes. We show that EBNA2 is sufficient to down regulation the HLA class II genes, and that this could be attributed largely to a down regulation of the master transcriptional regulator of HLA-class II CIITA [40]. EBNA2 binds to several regions near the CIITA gene, and we show that EBNA2 binding and activation of a neighboring gene correlates with inhibition of CIITA. We propose that EBNA2 inhibits CIITA transcription by a novel mechanism of enhancer competition across segregated chromosome domains.

## Results

### EBV down-regulates HLA class II genes during B-cell immortalization

**Fig 1:**
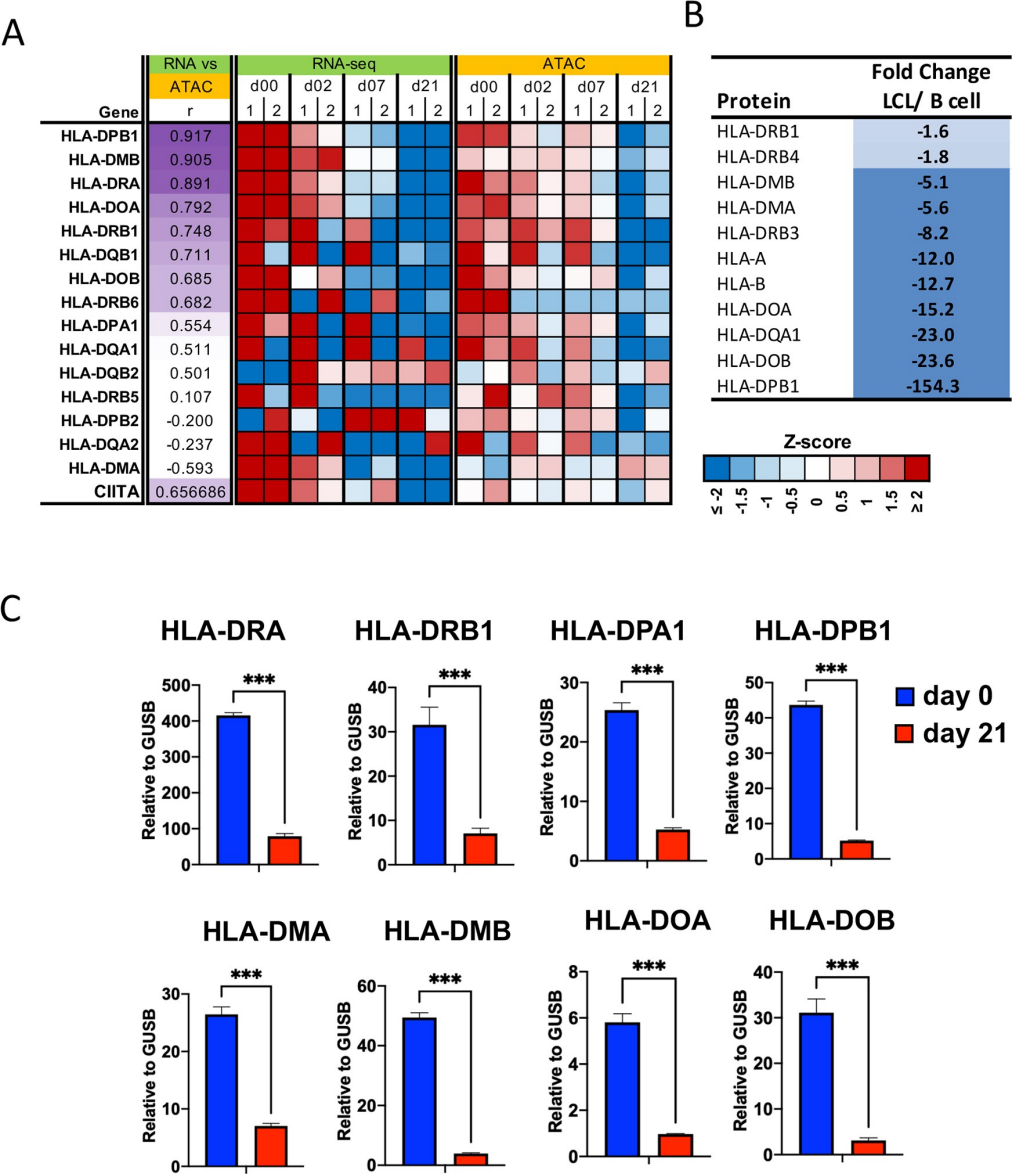
EBV represses transcription and chromatin of HLA class II gene cluster. **(A)** Heat map of RNA-seq and ATAC-Seq data from EBV infection of primary B-cells for two independent donors. HLA-II and CIITA genes are shown that have associated changes in both transcription levels and chromatin accessibility during primary infection (r > 0.5). The intensity of purple color for column r is proportional to correlation coefficient. Intensity for gene expression and ATAC signal is a z-score calculated among samples at each experiment (lower right). (**B**) Proteomic analysis of HLA protein changes in EBV infected LCLs relative to uninfected primary B-cells averaged for 2 donors using label-free quantification. Color is proportional to magnitude of fold change. (**C**) RT-qPCR analysis of HLA-DRA, -DRB1, -DPA1, -DPB1, -DMA, -DMB, -DOA, and -DOB in B cells (Day 0) and LCLs derived from the B cells (Day 21). Statistical analysis was performed in GraphPad Prism 9. Error bars are standard deviation from mean (SDM) and *** indicates p-values <0.001 using 2-tailed student t-test.

Examination of RNA-seq transcriptomic data from EBV infection of primary B-cells at different time points in the immortalization process revealed consistent pattern of down-regulation of HLA class II related genes (**Fig 1A**) [41]. The most significant changes were observed for HLA-DPB1, DMB, DRA, DOA, and these changes in RNA levels also correlated with changes in ATAC-seq peaks at these gene loci. Proteomics analysis also found a down-regulation of many HLA class II proteins (DPB1, DOB, DOA1), as well as some class I HLA protein (HLA-B, HLA-A), in EBV+ LCL compared to uninfected primary B-cells (**Fig 1B**). RT-qPCR confirmed that HLA-DRA, DRB1, DMA, DMB, DOA, DOB, DPA1, DPB1 transcripts were significantly decreased at day 21 post-EBV infection compared to day 0 (**Fig 1C**).

### EBNA2 is sufficient for down-regulation of HLA class II transcription

**Fig 2:**
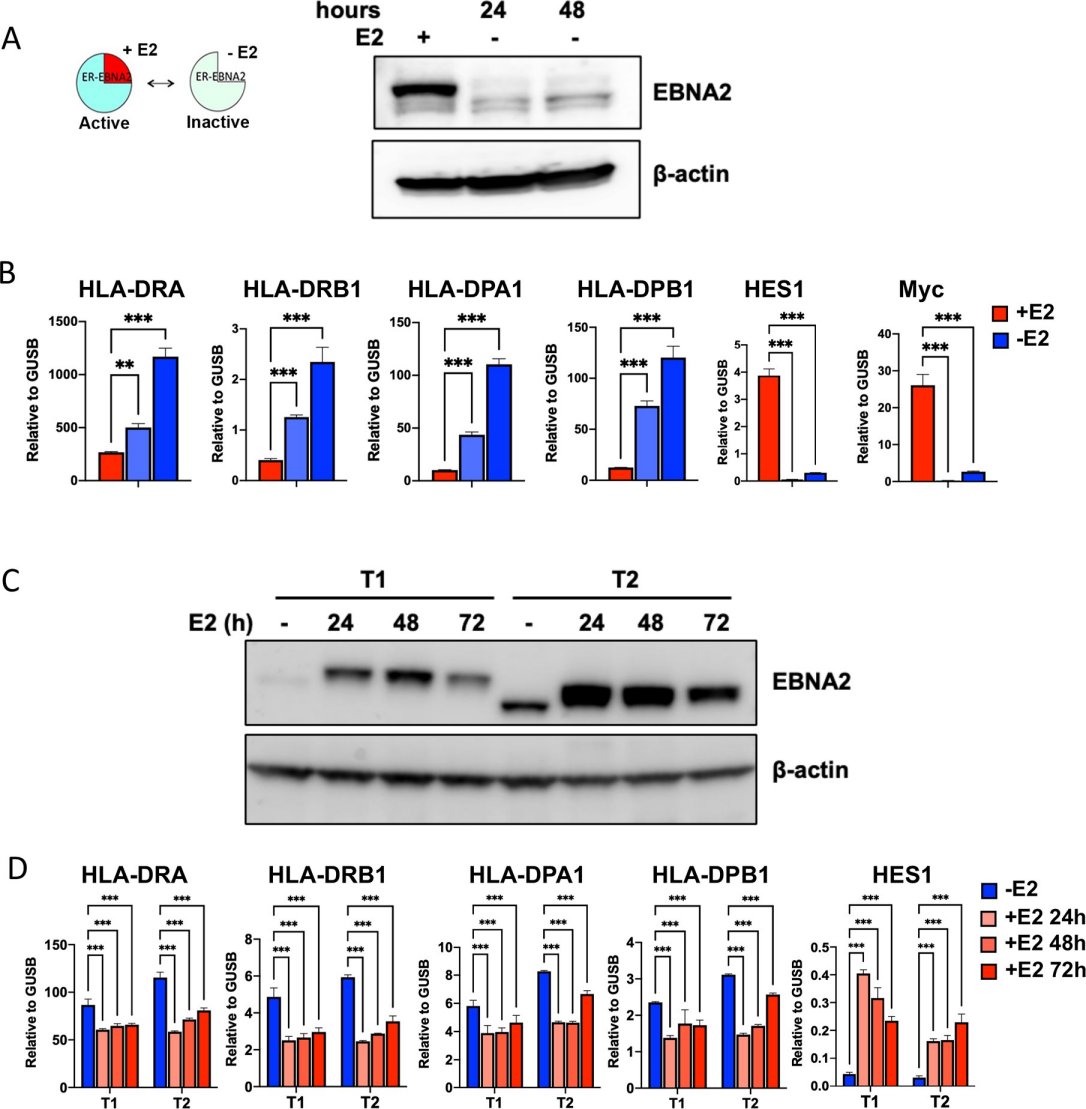
EBNA2 downregulates the expression of HLA-II. **(A-B)** EREB2.5 cells were treated with (+) or without (-) estradiol (E2) for 24 or 48 hrs and then assayed by Western blot for EBNA2 or loading control β-actin (**A**), and by RT-qPCR for HLA-DRA, -DRB1, -DPA1, -DPB1, or HES1 and c-Myc expression relative to GUSB (**B**). (**C-D**) Akata T1 or T2 cells were treated with (+) or without (-) estradiol (E2) for 24, 48 or 72 hrs and then assayed by Western blot for EBNA2 or loading control β-actin (**C**) or RT-qPCR for HLA-DRA, -DRB1, -DPA1, -DPB1, or HES1 expression relative to GUSB (**D**). Error bars are standard deviation from mean (SDM) and ** p<0.01, *** p <0.001 using 2-tailed student t-test.

EBNA2 is a potent regulator of host gene expression and essential for EBV immortalization in vitro [31]. Examination of published ChIP-Seq data sets revealed that EBNA2 bound to many sites in the HLA locus. To investigate the potential role of EBNA2 in regulating HLA gene expression, we tested whether conditional expression of EBNA2 modulates HLA class II gene transcription in latently infected lymphoblastoid cells. We first utilized EREB2.5 cell lines that express an estrogen receptor (ER)-EBNA2 fusion allowing conditional destabilization of EBNA2 protein upon withdrawal of estradiol (E2) [42] (**Fig 2A**). We found that inactivation of EBNA2 in EREB2.5 cells led to a significant increase in HLA class II gene transcription, as shown for DRA, DRB1, DPA1, DPB1 by RT-qPCR **(Fig 2B**). As a control, we show that addition of E2 did not alter HLA class II genes in normal LCLs with native EBNA2, indicating the effect of E2 on HLA expression is dependent on the EBNA2-ER fusion protein (**S1 Fig**). EBNA2 is well-known for its transcriptional activation of many cellular target genes, such as HES1 and c-myc [38,43]. As expected, and in contrast to HLA-class II gene increase, we observed a significant decrease in HES1 and myc transcription upon withdrawal of E2 and inactivation of EBNA2 (**Fig 2B**). Since EREB2.5 cells can also express other EBV gene products such as EBNA3C and LMP1, we tested the effect of conditional expression of EBNA2 in EBV negative Akata cells [44] (**Fig 2C**). We compared Akata cell lines with inducible EBNA2 from type 1 (T1) or type 2 (T2) EBV strains [44]. We found that both ER-activation of EBNA2 T1 and T2 led to a significant decrease in HLA-class II genes, while activating known target gene HES1 (**Fig 2D**). We did not observe any significant differences in EBNA2 T1 and T2 for repressing HLA-II genes.

### Down regulation of B-cell HLA-class II transcription correlates with decrease T-cell activation in mixed cell reactions

**Fig 3:**
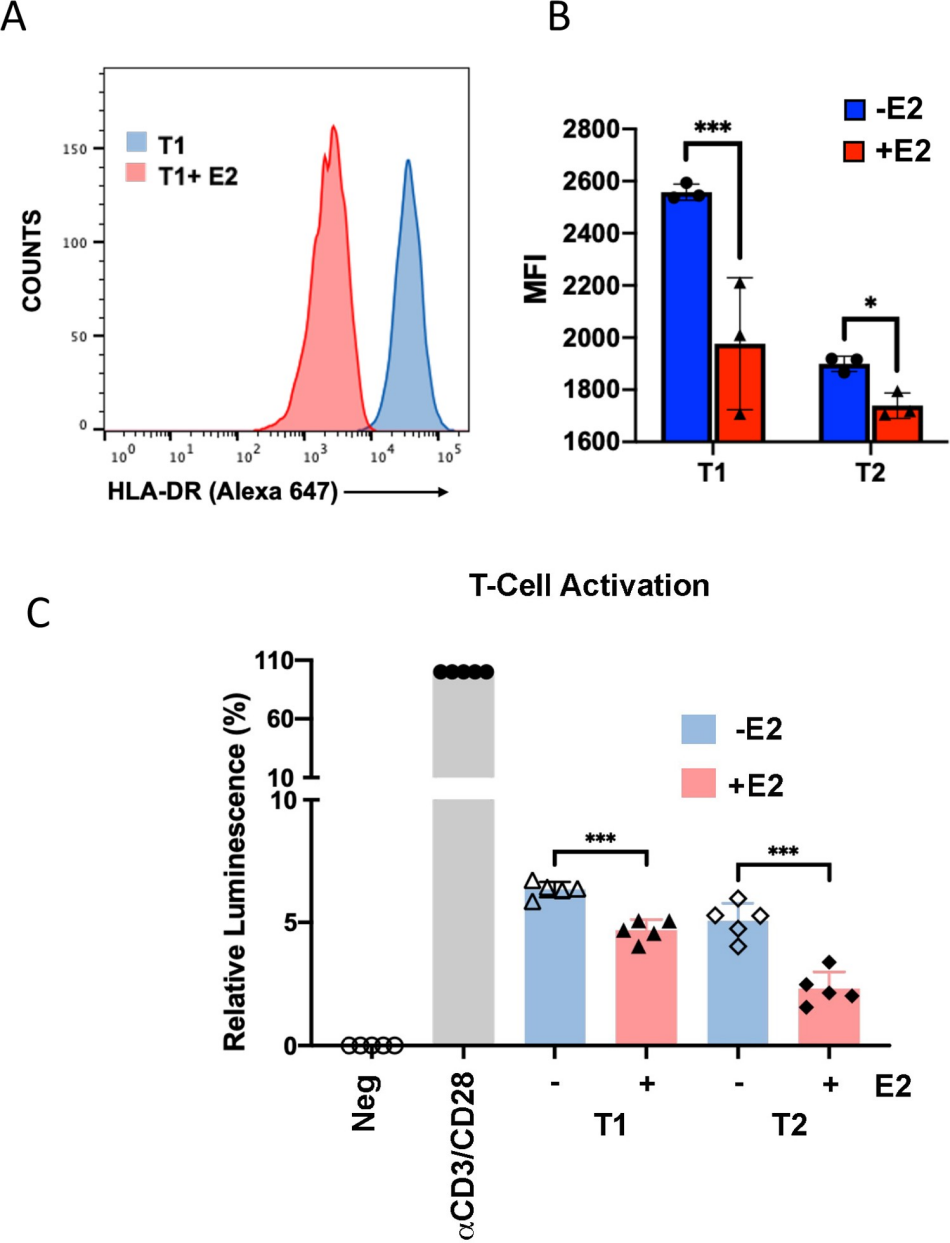
EBNA2 reduces HLA-II cell surface expression and stimulation of allogenic CD4+ T cells. **(A-B)** The surface expression of HLA-DR proteins on Akata T1 or T2 cells -/+E2 was determined by FACS. Representative FACS profile of Akata T1 -/+E2 (**A**) or mean fluorescence intensity values of Akata T1 or T2 cells -/+E2 (**B**) of surface HLA-DR expression are shown. Error bars are SDM and *p<0.05, *** p<0.001 using student 2-tailed t-test. (**C**) Freshly isolated human CD4+ T cells were incubated without stimulation as Negative (Neg) control, incubated with anti-CD3/CD28 beads as Positive (Pos) control, or cocultured with Akata T1 or T2 cells with (+) or without (-) estradiol (E2) and assayed for ATP levels by CellTiterGlo at day 6. Luminescence of Neg was set as 0, and Pos set as 100. Error bars are SDM, *** p<0.001 using 2-tailed student t-test.

To determine if the transcriptional down-regulation of HLA-II genes corresponds to a decrease in HLA protein expression on the cell surface, we assayed Akata EBNA2 (T1) cells by FACS (**Fig 3A and 3B**). FACS analysis of HLA-DR protein expression revealed a significant change in mean fluorescent intensity (MFI) in Akata EBNA2 (T1) cells treated with E2. Similar changes were observed with EBNA2 (T2) cells (**Fig 3B**). This demonstrates that EBNA2 expression correlates with a loss of cell surface HLA protein expression. The functionality of HLA expression was measured using a mixed lymphocyte reaction with allogenic CD4+ T cells (**Fig 3C**). Freshly isolated CD4+ T cells co-cultured with Akata T1 or T2 cells treated with (+) or without (-) estradiol and assayed by CellTiterGlo for T-cell activation (**Fig 3C**). We used anti-CD3/CD28 beads as a positive control. We found that Akata T1 and T2 cells treated with (+) estradiol were attenuated for T-cell activation. These findings suggest that EBNA2 mediated decrease in HLA-II cell surface expression also correlates with the loss of B-cell mediated T-cell activation.

### EBNA2 binds HLA-II locus and colocalizes with CIITA-bound enhancer elements

**Fig 4:**
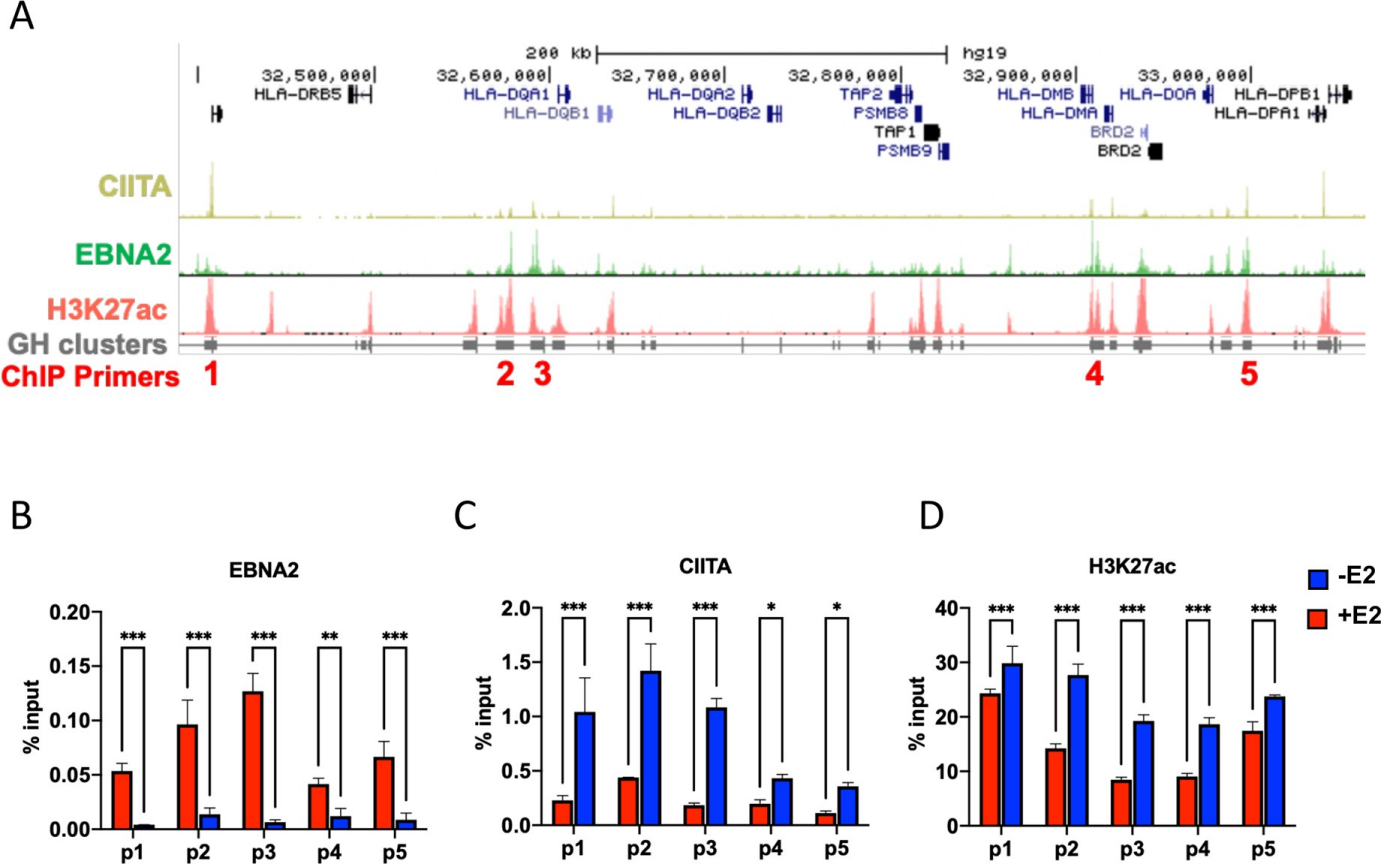
EBNA2 regulates the occupancy of CIITA and H3K27ac at HLA II region. **(A)** ChIP-seq tracks of CIITA, EBNA2, H3K27ac and GeneHancer clusters at HLA-II region using UCSC genome browser. ChIP primer positions p1-p5 are indicated. (**B-D**) ChIP-qPCR in EREB2.5 treated with (+) or without (-) estradiol (E2) with antibodies to either EBNA2 **(B**), CIITA (**C**), or H3K27ac (**D**) at primer positions p1-p5, as indicated in panel A. 2way ANOVA with Fisher’s Least Significant Difference (LSD) test was performed to assess significance. Error bars are SDM, and *p<0.05, **p<0.01, ***p<0.001 or ns (not significant).

Since the entire cluster of HLA II genes were down regulated by EBNA2, we suspected that these effects are mediated through the master transcriptional regulator of HLA-II CIITA [40]. Analysis of published ChIP-seq data revealed that EBNA2 and CIITA both bound to numerous sites across the HLA-class II region (**Fig 4A**). Analysis of published H3K27ac and Genehancer annotation revealed that EBNA2 and CIITA were enriched at enhancer elements throughout the HLA locus. To test the effects of conditional inactivation of EBNA2 on these enhancer elements, we assayed EBNA2, CIITA and H3K27ac by ChIP-qPCR at 5 enhancer positions (p1-p5) across the HLA class II locus in EREB2.5 cells with (+) or without (-) estradiol (E2) (**Fig 4B–4D**). As expected, E2 depletion caused the loss of EBNA2 binding at each of the primer positions (p1-p5) (**Fig 4B**). In contrast, E2 depletion led to an increase in CIITA (**Fig 4C**) and H3K27ac (**Fig 4D**) at each of the enhancer locations. These findings suggests that EBNA2 represses HLA-class II enhancer elements by restricting CIITA binding and H3K27ac formation.

### EBNA2 down regulates CIITA transcription and master regulator of HLA class II expression

**Fig 5:**
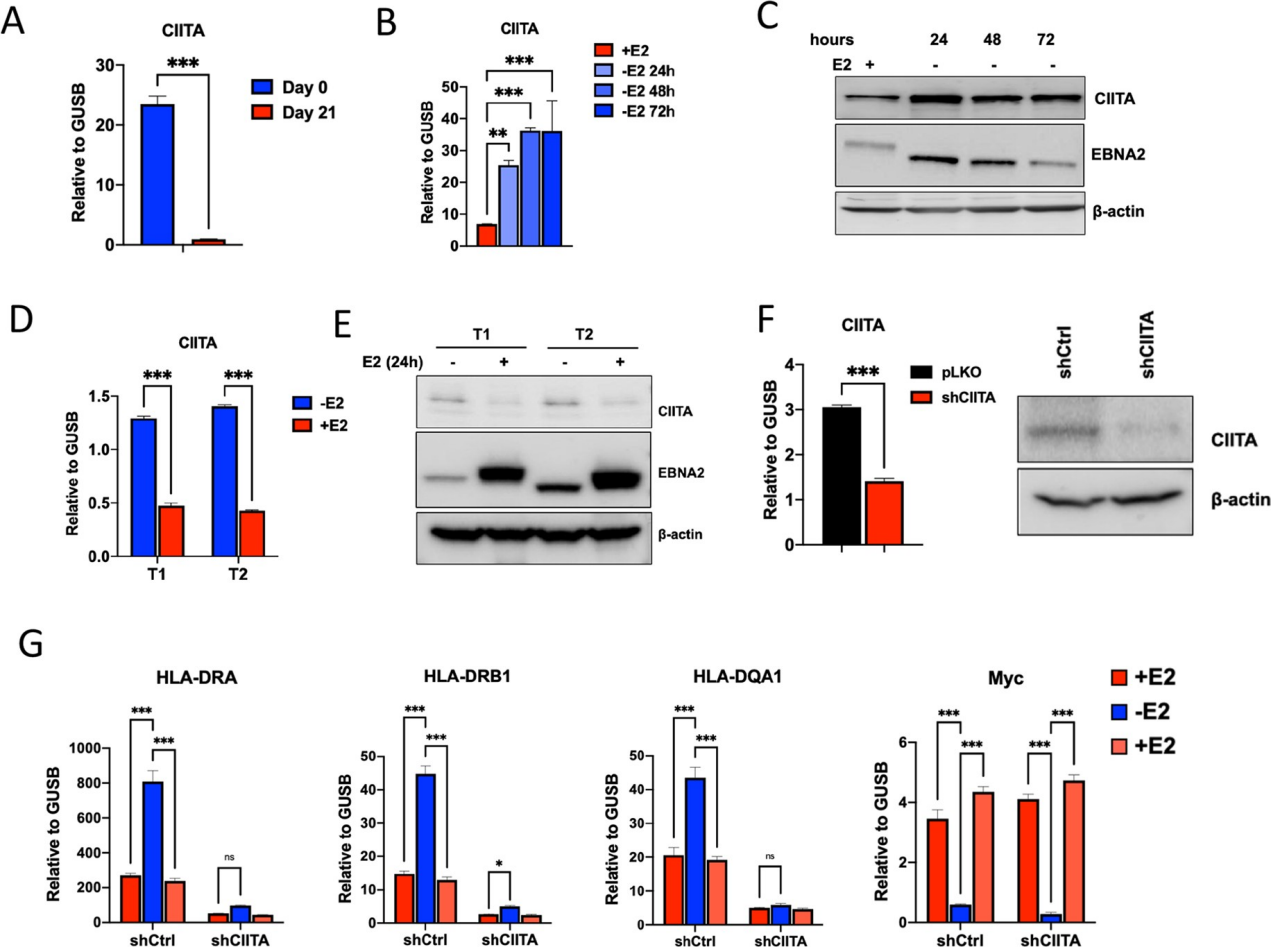
EBNA2 regulates HLA-II through CIITA. (**A**) RT-qPCR analysis of CIITA levels in B cells (Day 0) and LCLs derived from the B cells (Day 21). (**B-C**) EREB2.5 cells were treated with (+) or without (-) estradiol (E2) and then assayed by RT-qPCR for CIITA expression relative to GUSB (**B**) or Western blot for CIITA, EBNA2 or loading control β-actin (**C**). (**D-E**) Akata T1 or T2 cells were treated with (+) or without (-) estradiol (E2) and then assayed by RT-qPCR for CIITA expression relative to GUSB (**D**) or Western blot for CIITA, EBNA2 or loading control β-actin (**E**). (**F**) EREB2.5 transduced with either shCIITA or control pLKO.1 (shCtrl) lentivirus was assayed by RT-qPCR for CIITA relative to GUSB (left) or Western blot for CIITA and β-actin control (right). (**G**) Control or CIITA knockdown EREB2.5 cells were starved from E2 for 48hrs, then replenish with culture medium containing E2 and assayed by RT-qPCR for HLA-DRA, -DRB1, -DQA1 and Myc relative to GUSB. For A-F, 2-tailed student t-test was performed to determine the significance. For G, a 2way ANOVA with Fisher’s LSD test was performed to assess significance. Error bars are SDM, and * p<0.05, *** p<0.001 or ns (not significant).

Since CIITA binding was decreased at all positions in HLA-II locus, we next tested whether the expression of CIITA was decreased by EBV infection and, more specifically, EBNA2. RNA-seq transcriptomic indicated that CIITA is down regulated during EBV primary infection (**Fig 1A**), and we confirmed that by RT-qPCR comparing 21 day LCL to primary B-cells from the same donor (**Fig 5A**). We next tested whether EBNA2 was necessary for CIITA repression using the EREB2.5 system (**Fig 5B and 5C**) or sufficient using Akata cell system expressing inducible EBNA2 type 1 (T1) or type 2 (T2) (**Fig 5D and 5E**). We found that CIITA transcription and protein expression was significantly down regulated by EBNA2 in both EREB2.5, and Akata T1 and T2 cells (**Fig 5B–5E**). To determine whether the effect of EBNA2 repression of HLA-II genes was dependent on CIITA, we used lentivirus shRNA to deplete CIITA in EREB2.5 cell system. Depletion of CIITA was confirmed by RT-qPCR and Western blot (**Fig 5F**). In EREB2.5 cells transduced with control shRNA, withdrawal of E2 led to the expected increase in HLA-DRA, -DRB1, -DQA1, and decrease in myc, as well as the return to basal level upon re-addition of E2 (**Fig 5G**). In contrast, EREB cells transduced with shCIITA failed to activate HLA-DRA, -DRB1, -DQA1 upon E2 withdrawal. Importantly, CIITA depletion had no effect on EBNA2 activation of myc. These findings indicate that CIITA is required for EBNA2-dependent transcriptional regulation of HLA II genes.

### An EBNA2 binding site down-regulates CIITA and activates the neighboring DEXI gene

**Fig 6:**
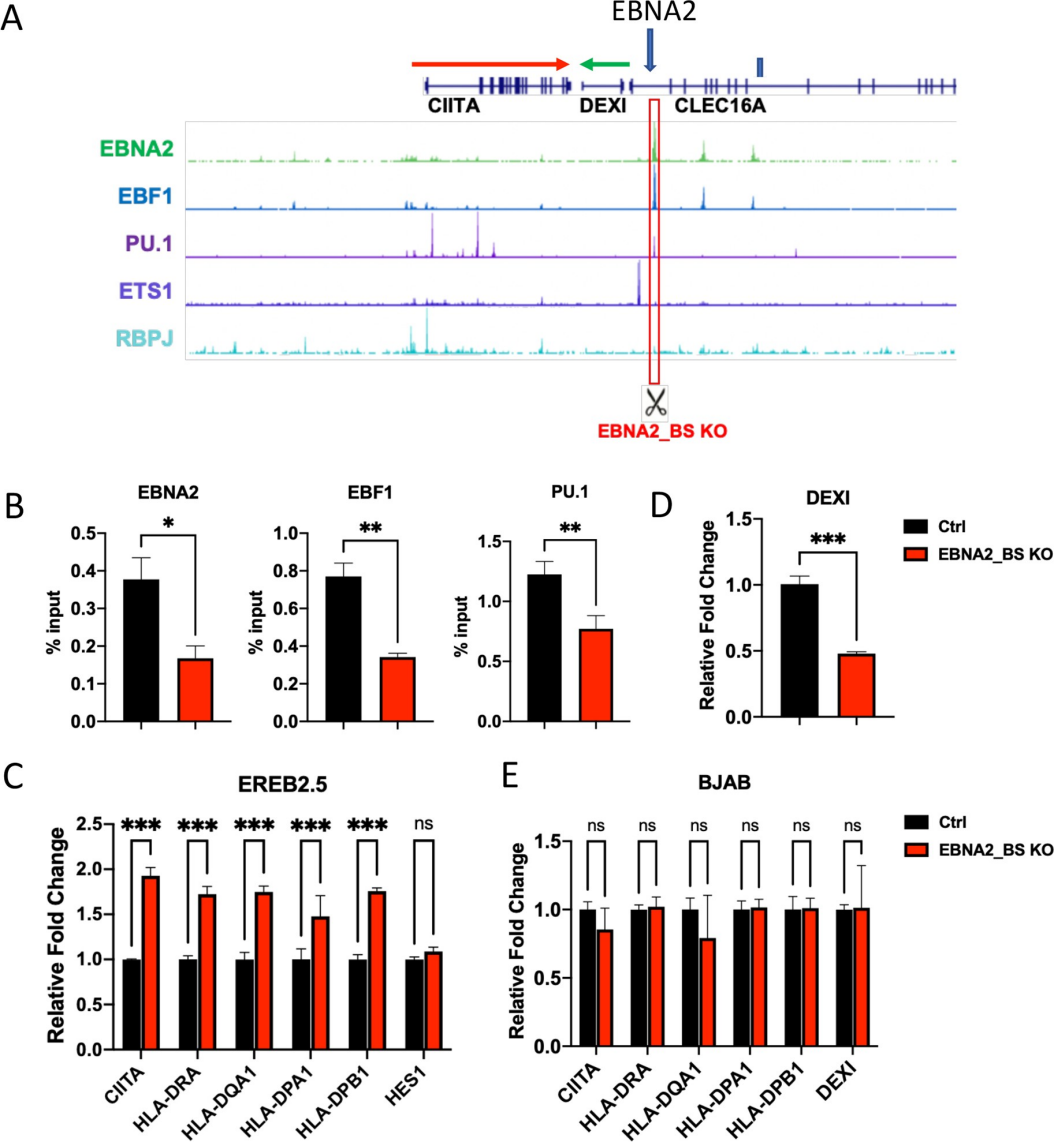
CRISPR mutated EBNA2 binding site alters CIITA and HLA-II gene expression. (**A)** Screenshot of UCSC genome browser with ChIP-seq tracks of EBNA2, EBF1, PU.1, ETS1, RBPJ and GeneHancer interactions at CIITA region. gRNA-targeted region is indicated by a red box. (**B**) ChIP-qPCR in Ctrl or EBNA2_BS KO EREB2.5 cells with antibodies to either EBNA2, EBF1, PU.1 or IgG. (**C)** Expression of CIITA, HLA-DRA, DQA1, DPA1, DPB1, and HES1 in Ctrl and EBNA2_BS KO EREB2.5 cells was measured by ∆∆CT method (2-tailed student t test; *** p<0.001 or ns (not significant)). (**D**) Same as in panel C showing DEXI gene only. **(E)** Expression of CIITA, HLA-DRA, DQA1, DPA1, DPB1, and DEXI in Ctrl and EBNA2_BS KO BJAB cells was measured by ∆∆CT method (2-tailed student t test; ns (not significant)).

To determine how EBNA2 may regulate CIITA, we first examined the published EBNA2 ChIP-seq data [36,45] and identified several candidate sites that are within ~100 kb of the CIITA TSS. We identified one major binding site located at the 3’ end of the CIITA gene transcript (**Fig 6A**). We used CRISPR/Cas9 gene editing to mutate the EBNA2 binding sites in LCLs. The EBNA2 binding site overlaps predicted binding sites for EBF1 and PU.1. Two independent pairs of guide RNAs (gRNAs) were designed to create ~200bp deletion at the EBNA2 binding site. We confirmed the deletion of the EBF1 and PU.1 binding site by PCR analysis of genomic DNA (**S2 Fig**). ChIP assay demonstrated that binding of EBF1, PU.1, and EBNA2 were significantly reduced in CRISPR edited LCLs (**Fig 6B**). We next assayed transcription in CRISPR EBNA2_BS ko vs control cells. We found that CRISPR EBNA2_BS ko cells had an increase in CIITA and HLA-DRA, DQA1, DPA1, DPB1 (**Fig 6C**). EBNA2 responsive gene HES1 was not affected by CRISPR ko of the EBNA2 binding site in CIITA locus. In contrast, the DEXI gene situated downstream and in the opposite orientation to CIITA was downregulated in cells lacking the EBNA2 binding site (**Fig 6D**). As a control, the same CRISPR ko was performed in BJAB cells, an EBV- and EBNA2-negative lymphoma cell and had no effect on CIITA, HLA-II, or DEXI gene transcription (**Fig 6E**). These findings suggest the EBNA2 binding site at the 3’ region of CIITA gene is important for the repression of CIITA and the activation of DEXI in EBV positive LCLs.

### EBNA2 binding selects RNAPII at DEXI promoter at expense of CIITA

**Fig 7:**
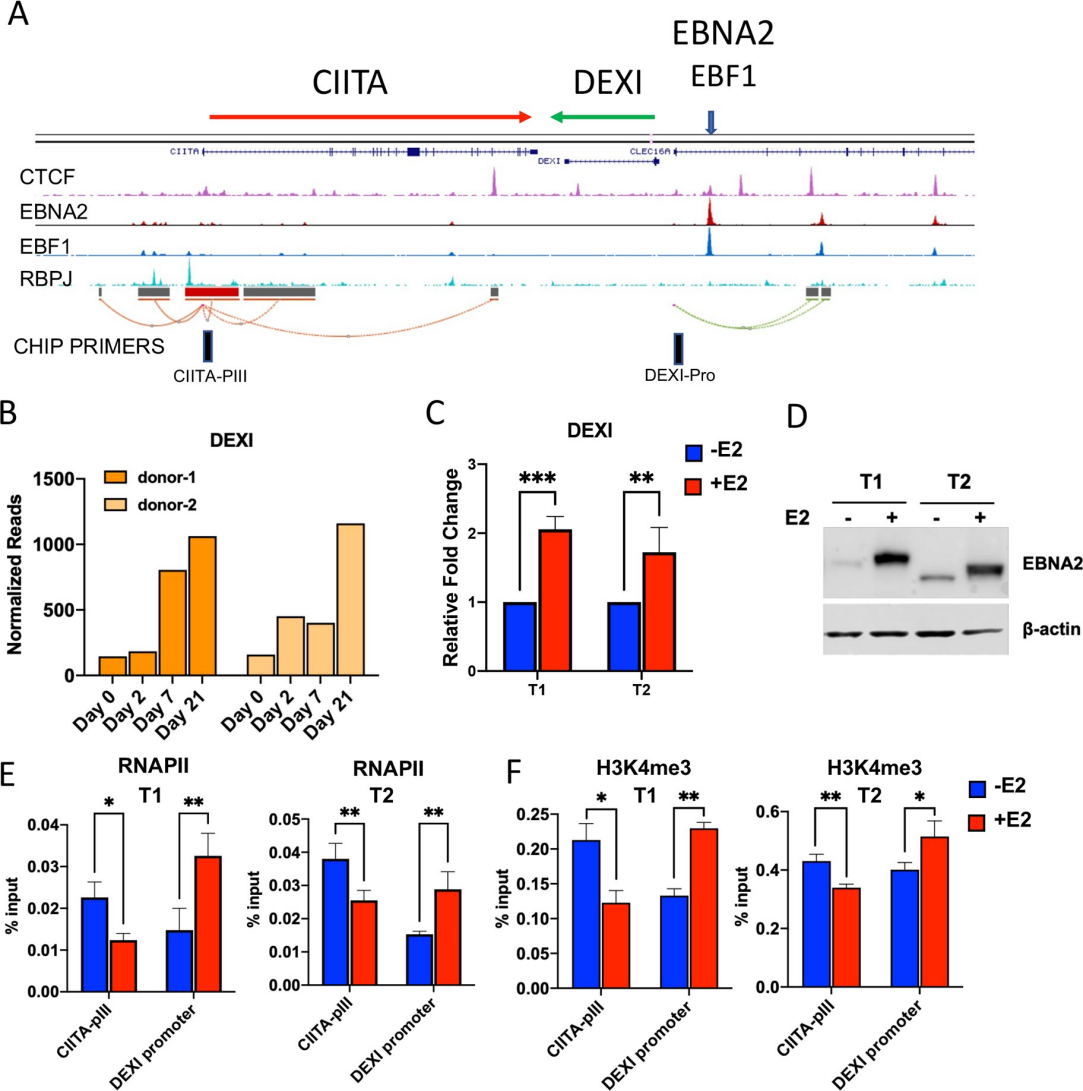
EBNA2 activates the neighboring DEXI gene. **(A)** ChIP-Seq for CTCF, EBNA2, EBF1 and RBPJ shown on UCSC browser. ChIP-primers position for CIITA-PIII and DEXI-promoter are indicated. **(B)** RNA-seq read-count quantification of DEXI transcripts during EBV infection of primary B-cells for 2 donors. **(C)** Akata T1 and T2 cells induced with estradiol for 48 hrs and assayed by RT-qPCR using the ∆∆CT method. **(D)** Western blot of EBNA2 expression in Akata T1 and T2 cells without (-) or with (+) E2 addition for 48 hrs. **(E)** RNAPII ChIP assay in Akata T1 or T2 cells with (+) or without (-) E2 induction at primer positions for CIITA-pIII or DEXI promoter. (**F**) Same as in panel E, except for H3K4me3 ChIP. Error bars are SDM, and * p<0.05, ** p<0.01, *** p < .001 or ns (not significant) by 2-tailed student t-test.

To further investigate the regulation of DEXI by EBNA2, we re-examined the gene organization for the CIITA and DEXI genes and the relative positions of their known promoter-enhancer elements (**Fig 7A**). DEXI is positioned in the opposite orientation and head-to-head with CIITA. We noted that several CTCF binding sites were located between the promoters of each gene. We next queried our RNA-seq data and found that DEXI is strongly induced during EBV immortalization of B-cells (**Fig 7B**). We also found that DEXI transcription was upregulated by EBNA2 expression in Akata T1 and T2 cells (**Fig 7C and 7D**). We next asked whether EBNA2 induction altered the relative binding of RNA polymerase II (RNAPII) at DEXI promoter relative to CIITA promoter III (CIITA-pIII) which drives the constitutive expression of CIITA in B cells [46]. We found that EBNA2 expression led to an increase in RNAPII at DEXI promoter, with a corresponding decrease in binding at the CIITA-pIII, in both Akata T1 and T2 cells (**Fig 7E**). Similarly, the histone modification H3K4me3 that is closely correlated with promoter activation was enriched at DEXI and depleted at CIITA-pIII (**Fig 7F**). These findings suggest that EBNA2 binding upstream of the DEXI promoter functions as a classical transcriptional activator and reorganizes RNAPII localization and orientation preference for DEXI at the expense of CIITA.

### Chromatin compartments insulated by CTCF separate EBNA2 activated DEXI from repressed CIITA

**Fig 8:**
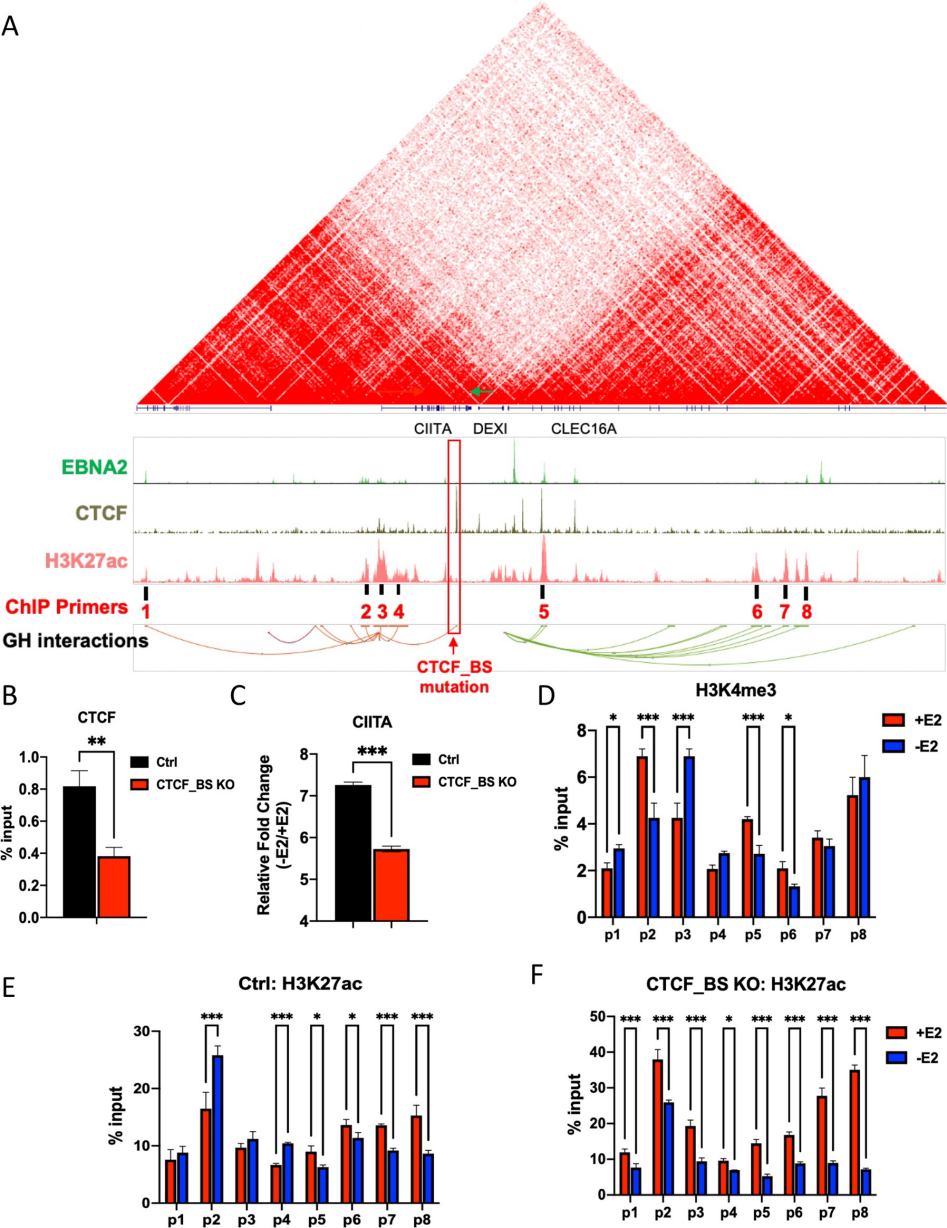
Chromatin boundary containing CTCF separates CIITA and DEXI gene. **(A)** HiC and ChIP-Seq for EBNA2, CTCF, and H3K27ac, and GeneHancer interactions shown on UCSC browser. CTCF binding site targeted by CRISPR and ChIP-primer positions p1-p8 are indicated. (**B**) CTCF ChIP assay in EREB2.5 CRISPR ctrl and binding site (BS) knock-out (ko). (**C**) CIITA fold changes in ctrl or CTCF BS ko cells with (+) or without (-) E2 were measured by ∆∆CT method (2-tailed student t test; *** p<0.001). (**D**) H3K4me3 ChIP-qPCR at positions p1-p8 in EREB2.5 cells. (**E and F**) H3K27ac ChIP-qPCR at positions p1-p8 in EREB2.5 CRISPR ctrl (**E**) and CRISPR CTCF BS ko cells (**F**) with (+) or without (-) E2 induction. For B-C, 2-tailed student t-test was performed to determine the significance. For D-F, a 2way ANOVA with Fisher’s LSD test was performed to assess significance. Error bars are SDM, and * p<0.05, *** p<0.001 or ns (not significant).

Analysis of published HiC data [47,48] suggests that CIITA promoter is in a different chromosome topological-associated domain (TAD) relative to the DEXI promoter, and that a strong chromosome boundary, as measured by HiC interactions, exists between these two regulatory domains **(Fig 8A**). To investigate whether chromatin boundary factors may contribute to the down regulation of CIITA by EBNA2 binding to a neighboring compartment, we analyzed ChIP-seq data for CTCF binding sites between CIITA and DEXI (**Fig 8A**). We used CRISPR/Cas9 gene editing to delete the prominent CTCF binding site between DEXI and CIITA using EREB2.5 cell system (**Fig 8A**). We confirmed the mutation by Sanger sequencing and TIDE analysis (**S3 Fig**). We found that the deletion caused a significant (~50%) decrease in CTCF binding (**Fig 8B**). We then assayed the relative expression of CIITA in response to EBNA2 in either control or CTCF binding site deleted cells (**Fig 8C**). We found that CTCF binding site deleted cells had diminished CIITA gene transcription relative to control cells after E2 withdrawal. The distribution of H3K4me3 and H3K27ac was assayed at various regions (p1-p8) across the CIITA-DEXI locus by ChIP-qPCR (**Fig 8D–8F**). E2 withdrawal led to a decrease in H3K4me3 at the EBNA2 bound enhancer-element for CIITA (primer position p2), and an increase in H3K4me3 at the CIITA promoter region (primer p3), correlating with EBNA2 repression of CIITA. Similarly, E2 withdrawal led to a decrease in H3K4me3 at the enhancer elements upstream of the DEXI promoter (p5, p6) (**Fig 8D**). The enhancer mark H3K27ac increased at the enhancer elements associated with CIITA binding (p2, p4) and decreased at multiple enhancer elements (p5, p6, p7, p8) upstream of DEXI in response to E2 withdrawal (**Fig 8E**). In CTCF binding site deleted cells (CTCF_BS KO) the pattern of H3K27ac showed a different response to E2 withdrawal than control cells, especially at the CIITA enhancer (p2) and promoter (p3) elements (**Fig 8F**). In CTCF_BS KO cells, the p2 and p3 enhancers of CIITA responded in the same direction as the DEXI enhancer elements. These findings indicate that CTCF binding site in the boundary between these two chromosome compartments is important for confining the activity of EBNA2 to one compartment at the expense of a neighboring compartment.

## Discussion

Diverse mechanisms have been described for the down regulation of HLA expression by pathogens and cancer cells. Here, we describe a novel and unusually indirect mechanisms of transcriptional down regulation of HLA-II genes by the EBV encoded transcriptional regulator EBNA2. EBNA2 typically functions as a potent transcriptional activator through the assembly and strengthening of enhancer-promoter interactions [34,38]. We show here the EBNA2 down-regulates HLA-II genes largely through the down regulation of CIITA transcription. Further, we show that EBNA2 down regulates CIITA by competitive activation of a downstream gene enhancer that sequesters RNA polymerase II to a neighboring and insulated chromosome compartment.

**Fig 9:**
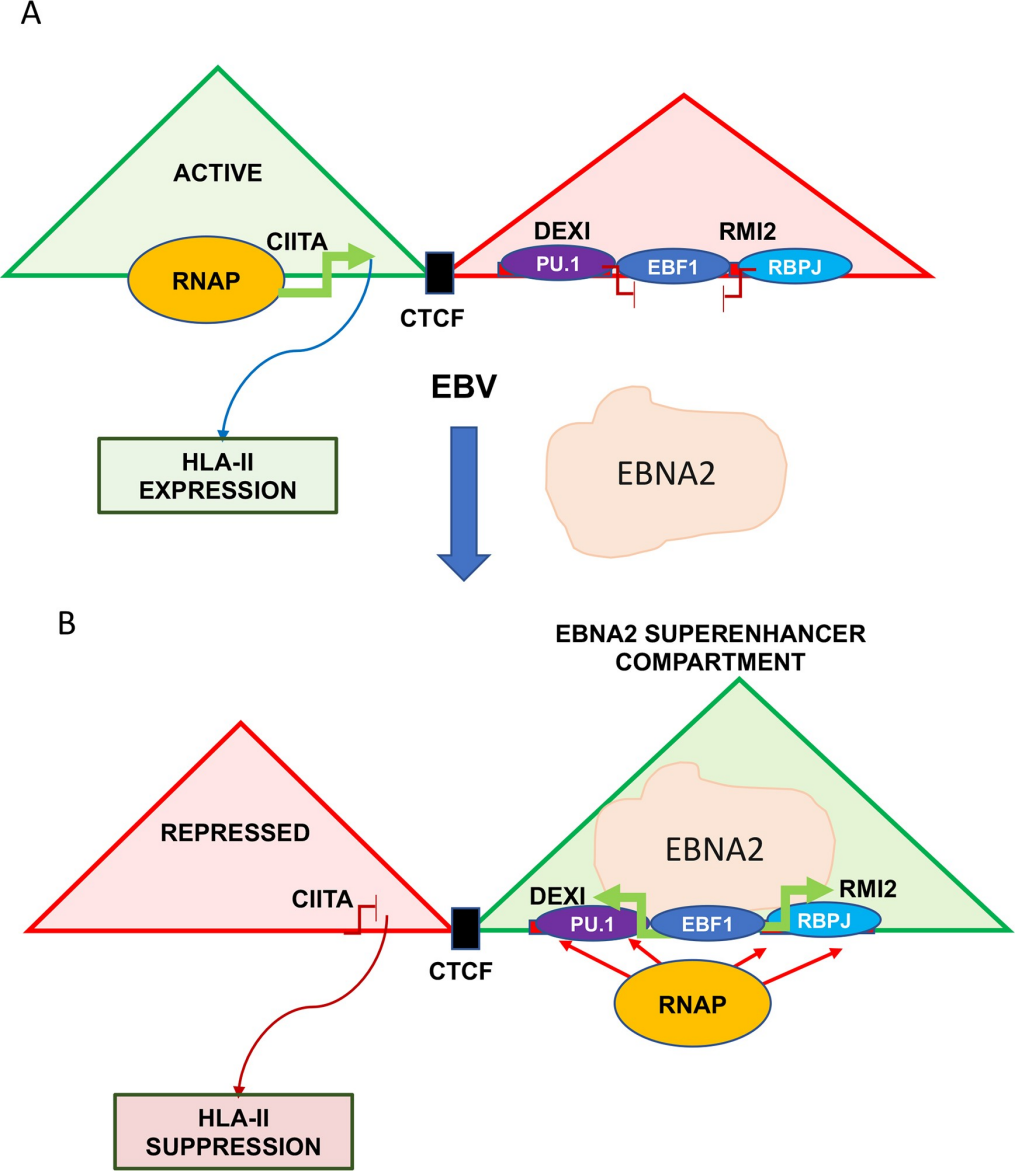
Model of EBNA2 indirect repression of HLA-II genes through down-regulation of CIITA using mechanism of enhancer compartment competition. In absence of EBNA2, CIITA enhancer is active and HLA-II gene expression is high. In presence of EBNA2, DEXI enhancer is activated, competing with CIITA promoter situated in a neighboring and bounded chromosome compartment. EBNA2 decrease of CIITA causes decrease in HLA-II expression.

EBV primary infection and B-cell immortalization leads to the down regulation of most HLA class II gene transcripts. We show that this correlates with a loss of HLA class II protein expression on the cell surface and loss of T-cell stimulatory activity. The down regulation of HLA-class II was dependent on EBNA2 expression and could be recapitulated in cells that conditionally express EBNA2 as the only induced viral protein. We investigated a potential direct mechanism and found that EBNA2 bound directly to multiple sites within the HLA locus and colocalized partly with CIITA binding sites. This suggests that EBNA2 may also antagonize CIITA transcriptional co-activator function at the HLA locus. However, we were unable to demonstrate a direct effect of EBNA2 at the HLA-II locus, nor any interaction between EBNA2 and CIITA. However, knock-down of CIITA indicated that EBNA2 regulation of HLA-II depends strongly on CIITA. We therefore investigated the indirect mechanism of EBNA2 regulating HLA through transcriptional regulation of CIITA. We found that EBNA2 localizes to a position downstream of the CIITA gene, and activates a downstream enhancer for another gene, DEXI, that is oriented in the opposite direction and head-to head with the CIITA transcript. Activation of DEXI by EBNA2 occurred through conventional increase in H3K27ac at the enhancer and H3K4me3 and RNAPII at the promoter region. In contrast, EBNA2 induced the opposite effect at the CIITA enhancer and promoter regions, corresponding to a decrease in CIITA transcription. Analysis of the chromosome domain structure revealed by HiC in EBV+ LCLs suggests that DEXI and CIITA enhancers are in different, but neighboring TADs, and that a strong boundary exists between these gene enhancers. Mutation of a CTCF site in the boundary region altered the effect of EBNA2 on the CIITA enhancers, causing them to respond similar to the DEXI enhancer. These findings suggest that CTCF segregates EBNA2 target genes, and that EBNA2 can repress some target genes through a competition with neighboring enhancers and chromosome compartments for RNAPII (**Fig 9**).

Regulation of CIITA is known to be complex and critical for HLA gene expression. CIITA was identified as a transcriptional coactivator essential for expression of HLA-class II genes. Its expression correlates tightly with HLA-II gene transcription, and is mostly restricted to lymphoid cells [40]. CIITA is known to be regulated through multiple cell-type specific promoter and distal enhancers that make a network of contacts with each promoter [40,49]. Several cellular factors have been implicated in CIITA regulation, including chromatin remodeling factors BRG1, histone acetylase p300, transcription factor STAT1 and IRF1 [49], and the polycomb repressive complex 2 (PRC2) [50]. EBNA2 repression of CIITA is likely to be through rewiring of local enhancer utilization. EBNA2 affected histone modifications at the CIITA promoter and enhancer with no evidence of its directly binding to these locations. Our findings suggest that EBNA2 activates the neighboring chromosome compartment, containing DEXI among other genes that are upregulated by EBV infection and EBNA2, including RMI2 and SOCS1. DEXI is a dexamethasone-responsive gene that has no known function. Another neighboring gene, CLEC16A, is a C-type lectin that has been implicated in BCR-dependent HLA-II pathway, and within the susceptibility locus for multiple sclerosis (MS) [51]. However, we did not observe any effect of EBNA2 on CLEC16A transcription, and the CRISPR deletion of a single CTCF binding sites between these two gene loci had only a small effect of EBNA2 regulation.

Although EBNA2 is thought to function predominantly as a transcriptional coactivator, we did find that EBNA2 colocalized at some CIITA sites in the HLA locus. We were unable to show any direct physical association of EBNA2 with CIITA by CoIP, but it is possible that EBNA2 and CIITA compete for occupancy or that EBNA2 modulates CIITA function at the HLA-II regulatory elements. While EBNA2 is not thought to be a transcriptional repressor, it is known to interact with the co-repressor BS69 through the MYND domain [52]. Type 2 EBNA2 interacts more strongly with BS69 and this correlates with its reduced capacity to activate LMP1 and transform B-cells [52]. Whether BS69 or other co-repressors interact with EBNA2 at the HLA or CIITA gene to confer transcriptional repression remains a potential alternative or additional mechanism to explain EBNA2 directed transcriptional repression.

Numerous pathogens are known to regulate HLA-II through CIITA expression and function. HIV is known to down-regulate HLA and TAT has been shown to antagonize CIITA transcription co-activator function [53,54]. CMV has been shown to down regulate HLA-II through a decrease in CIITA transcripts [55,56], through a mechanism involving the disabling of interferon-γ signaling through STAT1 [57,58]. Similar mechanisms of regulation have been observed with VZV infection [59,60]. KSHV down regulates MHC-II through viral IRF3 and SOCS2 mediated down regulation of CIITA [61]. KSHV LANA can also inhibit the PIII and PIV promoter of the CIITA gene to down regulate HLA-II expression [62]. Several different EBV proteins have been implicated in regulation of CIITA. EBV LMP2A suppressed class II expression by down-regulating the CIITA PIII promoter through the down regulation of B-cell transcription factors E47 and PU.1 [63]. EBV lytic activator ZTA has been shown to down regulate HLA-II through repression of the PIII promoter [24,25]. Like EBNA2, ZTA is typically associated with transcription activation, and the mechanism of transcriptional repression at CIITA was found to be independent of ZTA dimerization domain, and presumably DNA binding [24].

HLA class II gene expression is tightly controlled by CIITA and is highly relevant to B-cell development, antigen processing, and malignancies. HLA class II is frequently reduced in EBV+ B-cell malignancies, particularly HLA-DM in Hodgkin’s lymphoma [64,65]. Down-regulation of HLA may correspond to the natural development of memory B-cells, transition from GC to memory. EBNA2 has been shown to negatively regulate genes important for germinal center reaction, including TCL1 and CIITA using a DLBCL model [39], but a specific mechanism of transcriptional repression was not described. EBNA2 and EBF1 may also be responsible for the down regulation of CD74, which is an MHC II chaperone that facilitates MHC II folding and peptide binding in the ER [66]. Antigen processing proteases are different in EBV LCL compared to primary B-cells or endogenous activated B-cells in vivo [66], suggesting many aspects of HLA regulation are altered by EBV infection. Although EBV typically down-regulates HLA, a series of studies have found that EBV+ gastric carcinoma expressed higher levels of HLA class II gene relative to normal control gastric cells [67,68]. This correlated with an increase in CIITA and RFX5 expression [69]. HLA-II is up regulated in EBVaGC, where EBNA2 is not typically expressed [69]. This is also different than the effects observed in an EBV+ nasopharyngeal carcinoma, where HLA class II expression is down in ~50% of the cases [70,71]. Thus, EBNA2 down regulation of CIITA may be cell-type specific, depending on chromatin domain structure and enhancer wiring particular to B-cells but perhaps different in EBV epithelial cancers.

## Materials and methods

### Cell lines

Primary B cells were grown in B-cell medium (RPMI supplemented with 20% fetal bovine serum (FBS), 10mM HEPES, 1X Glutamax, and penicillin/streptomycin), and maintained in this medium for a minimum of 4 weeks (or until collected) after infection with EBV derived from Mutu I strain. Once proliferation was stabilized cells were transferred to RPMI medium supplemented with 10% FBS and antibiotics. Previously established LCLs and BJAB cells were also maintained in RPMI with 10% FBS and antibiotics. 293T cells (ATCC) were grown in DMEM with 10% FBS and antibiotics (penicillin and streptomycin).

EREB 2.5 is a lymphoblastoid cell line expressing the estrogen-inducible EBNA2-estrogen receptor (ER) fusion protein complementing P3HR1 EBV strain [72]. EREB2.5 was maintained in RPMI containing 10% FBS, antibiotics (penicillin and streptomycin), and 1μM estradiol (E2). Akata T1 and T2 cells are derived from EBV negative Akata Burkitt lymphoma cell line transfected with *oriP* episome expressing EBNA1 and estrogen-receptor fusion of EBNA2 for conditional activation of EBNA2 in the presence of estradiol [73]. Akata T1 and T2 cells were maintained in RPMI containing 10% FBS, antibiotics (penicillin and streptomycin), G418 (1 mg/ml), and puromycin (1 ug/ml). For estrogen starvation of EREB2.5 cells, the cells were washed twice in serum free RPMI, and resuspended in RPMI medium without estrogen for indicated time.

### Primary B-cell isolation and EBV infection

All infection studies were performed with deidentified human B-lymphocytes isolated from whole blood based on a modified protocol utilizing lymphocyte separation medium (Lymphoprep, STEMCELL Technologies) and specialized centrifugation tubes (SepMate-50, STEMCELL Technologies) [41]. For RNA-Seq and ATAC-Seq, purified B cells were resuspended in B cell medium, counted, and infected immediately after purification. EBV virus was concentrated by ultracentrifugation from stimulated Mutu I cells. EBV was added at an MOI of 1 and monitored by the growth and clumping of cells, a characteristic of lymphoblastoid cell lines [41]. RT-qPCR was performed as described previously [41], and primers are listed in **S1 Table.**

### Proteomics

For proteomic study, deidentified human B cells were obtained from the Human Immunology Core of the University of Pennsylvania under an Institutional Review Board-approved protocol, as previously described [74]. Briefly, primary B cells (25 x 10^6^ for each donor) were purified from donor plasma using the RosetteSep human B-cell enrichment cocktail (StemCell Technologies) and cultured in RPMI 1640 supplemented with fetal bovine serum (FBS, final concentration 15%), and 1% penicillin/streptomycin cocktail. EBV (B95.8 strain) was collected from supernatant of the EBV-positive ATCC cell line VR-1492TM and concentrated with the PEG virus precipitation kit (Abcam). 24 hrs after their collection, 20 x 10^6^ primary B cells were harvested for the assay, whereas 5 x 10^6^ were infected with the concentrated EBV. Primary infected B cells were weekly monitored during the EBV-induced transformation and cultured for 35 days post-infection (d.p.i.) before being considered lymphoblastoid cell lines (LCL). At 36 d.p.i., LCLs were harvested and processed for the proteomic assay together with their matched primary B cells. Cell lysates (25 μg each) were run into a NuPAGE 10% Bis-Tris gel (Thermo Scientific) for a short distance, and the entire gel lanes were excised and digested with trypsin. Liquid chromatography-tandem mass spectrometry (LC-MS/MS) analysis was performed using a Q Exactive HF mass spectrometer (Thermo Scientific) coupled with an UltiMate 3000 nano UPLC system (Thermo Scientific). Samples were injected onto a PepMap100 trap column (0.3 x 5 mm packed with 5 μm C18 resin; Thermo Scientific), and peptides were separated by reversed phase HPLC on a BEH C18 nanocapillary analytical column (75 μm i.d. x 25 cm, 1.7 μm particle size; Waters) using a 4-h gradient formed by solvent A (0.1% formic acid in water) and solvent B (0.1% formic acid in acetonitrile). Eluted peptides were analyzed by the mass spectrometer set to repetitively scan m/z from 400 to 2000 in positive ion mode. The full MS scan was collected at 60,000 resolution followed by data-dependent MS/MS scans at 15,000 resolution on the 20 most abundant ions exceeding a minimum threshold of 20,000. Peptide match was set as preferred, exclude isotope option and charge-state screening were enabled to reject unassigned and single charged ions. Peptide sequences were identified using MaxQuant 1.6.17.0 [75]. MS/MS spectra were searched against the UniProt human protein database and a common contaminants database using full tryptic specificity with up to two missed cleavages, static carboxamidomethylation of Cys, and variable Met oxidation, protein N-terminal acetylation and Asn deamidation. “Match between runs” feature was used to help transfer identifications across experiments to minimize missing values. Consensus identification lists were generated with false discovery rates set at 1% for protein and peptide identifications. Protein fold changes were determined from the LFQ intensity. Missing values were imputed with the minimum LFQ value, and t-test p-values were adjusted to account for multiple testing using Benjamini-Hochberg FDR.

### Mixed lymphocyte reactions

PBMCs were separated from whole blood as described above, and CD4 T cells were isolated using Dynabeads Untouched Human CD4 T cells kit following the manufacturer’s protocol (Invitrogen). CD4 T cells were diluted such that 25,000 cells were added per 100 μl in each well of a 96-well plate. Akata T1 and T2 cells were treated with 50 μg/ml mitomycin C (Sigma-Aldrich) for 20 min at 37°C, washed three times, and diluted such that 5,000 cells were added per 100 μl in each well of a 96-well plate. The use of mitomycin C prevented Akata T1 and T2 from overgrowing the culture. CD4 T cells were cultured alone (Negative), with anti-CD3/ CD28 Dynabeads Human T-Activator (Thermo Fisher Scientific) (Positive), or with 5,000 Akata T1 or T2 cells in RPMI medium -/+ estradiol for 6 days. Cell viability was determined using the CellTiter-Glo assay (Promega) at day 6.

### Western blot

Equal amounts of protein extract in RIPA buffer (50mM Tris-HCl (pH8.0); 150mM NaCl; 1% NP-40; 0.5% Sodium deoxycholate; 0.1% SDS; 1mM EDTA) were resolved in 8–16% Novex Tris-Glycine gels (Invitrogen), and then transferred onto a PVDF membrane (Millipore), where they were incubated with specific antibodies followed by HRP-conjugated secondary antibodies (BioRad) and ECL reagents (Millipore) for detection.

### Chromatin Immunoprecipitation (ChIP)

Cells were crosslinked in 1% formaldehyde for 15 min, followed by quenching for 5 min with 0.125 M glycine. 1 × 10^7^ cells were lysed in 1 ml SDS lysis buffer (1% SDS, 10 mM EDTA, and 50 mM Tris-HCl, pH 8.0) containing 1 mM PMSF and protease inhibitor cocktails (Sigma-Aldrich), and kept on ice for 10 min. Lysates were sonicated with a Diagenode Bioruptor, cleared by centrifugation to remove insoluble materials, and diluted 10-fold into IP Buffer (0.01% SDS, 1.1% Triton X-100, 1.2mM EDTA, 16.7mM Tris pH 8.0, 167mM NaCl, 1 mM PMSF, and protease inhibitors cocktail), and incubated with anti-H3K27ac (Abcam, ab4729), anti-H3K4me3 (Millipore, 07473), anti-EBNA2 [PE2] (Abcam, ab90543), anti-EBF1 (Millipore, AB10523), anti-PU.1 (Millipore, 04–1072), anti-CTCF (Millipore, 07–729), anti-RNAPII (Abcam, ab817), or anti-CIITA (Rockland Immunochemicals Inc., 100-401-249) overnight at 4°C. Preblocked protein A/G sepharose (GE Healthcare, 17-0780-01/17-0618-01) was added to each IP reaction for additional 2 to 3 h incubation at 4°C. Each immune complex was washed five times (10 min each) in ChIP related wash buffer at 4°C, and eluted with 150 μl Elution buffer (10mM Tris, pH 8.0, 5mM EDTA, and 1% SDS) at 65°C for 30 min. The elutes were then incubated at 65°C overnight to reverse cross-linking, and further treated with Proteinase K in a final concentration of 100 μg/ml at 50°C for 2 hrs. ChIP DNA was purified by Quick PCR Purification Kit (Life Technologies) following the manufacturer’s instruction. ChIP DNA was assayed by qPCR using primers specific for indicated regions and quantified as % input. Primers for ChIP-qPCR are listed in **S2 Table**.

### Lentiviral transduction

pLKO.1 vector-based shRNA constructs for CIITA (TRCN0000019072) were obtained from Open Biosystems. Control shRNAs (shControl or shCtrl) were generated in the pLKO.1 vector with the target sequence 5′-TTATCGCGCATATCACGCG-3′. Lentiviruses were produced in 293T cells using envelope and packaging vectors pMD2.G and pSPAX2 as described previously. EREB2.5 cells were infected with lentiviruses carrying pLKO.1-puro vectors by spin infection at 450 × g for 90 min at room temperature. The cell pellets were resuspended and incubated in fresh RPMI medium (containing 1μM estradiol) and then treated with 2 μg/ml puromycin at 48 h after the infection. The RPMI medium with 2μg/ml puromycin was replaced every 2 to 3 days. The cells were collected after 7 days of puromycin selection and then subjected to further analyses, as indicated.

### CRISPR/Cas9 mutagenesis

gRNAs targeting CTCF binding motif or EBNA2 binding site were cloned into the lentiCRISPRv2 (Addgene 52961) using published protocols [76]. Lentiviruses were produced and EREB2.5 cells were transduced as described above. Genomic DNA from cells was isolated with Genomic DNA purification kit (Promega), gRNA target sites were amplified by PCR, analyzed by agarose gel electrophoresis, and the PCR products were Sanger sequenced at Wistar Institute sequencing facility. The sequence trace was analyzed by the TIDE algorithm (available at https://tide.nki.nl) [77].

### Flow cytometry

Cells were harvested, washed twice with ice cold FACS buffer (PBS with 5% FBS and 0.1% NaN3), and resuspended at 2.5x10^6^ cells/ml in the same buffer. Cells were blocked with Fc block for 20 min on ice followed by staining of surface-expressed HLA-DR at room temperature for 30 min with anti-HLA-DR (Abcam, ab136320). Then the cells were washed three times with FACS buffer, incubated in the dark with secondary antibody (anti-mouse ALEXA Fluor 647, Abcam ab150107) for additional 30 min at room temperature, washed three times and resuspended in ice cold FACS buffer. Then flow cytometric data were collected on an LSRII and analysis of flow cytometry data was conducted with FlowJo. The experiment was conducted in triplicate.

### Next-generation sequence data

RNA-Seq and ATAC-Seq on EBV infected B-cells was described previously[41]. HiC data was extracted from published dataset for LCL [48]. Data sets from NCBI GEO are H3K27ac (GSM733771), PU.1 (GSM803531), ETS1 (GSM803510), CIITA (GSM1602235), EBNA2 (GSE47629), EBF1 (GSM1958039), RBPJ (GSM1958041), and CTCF (GSM3720519).

## Supporting information

S1 Fig Estradiol does not alter MHC II expression in LCL. To rule out the potential impact of estradiol on HLA transcription, LCL352 was treated with (+) or without (-) estradiol for 48 hrs and then assayed by RT-qPCR for HLA-II gene transcription.
(TIF)

S2 Fig Validation of EBNA2_BS CRISPR knock out in EREB2.5 and BJAB. (**A**) Schematic diagram showing gRNA positions and location of EBF1 and PU.1 motifs. (**B**) After CRISPR deletion of EBNA2_BS, genomic DNA was PCR amplified and verified for deletion efficiency.
(TIF)

S3 Fig TIDE analysis of CTCF binding site CRISPR knock out. To confirm the CTCF-BS mutation, PCR was performed to amplify the gRNA targeted region. The purified PCR fragments were Sanger sequenced (A) and analyzed using TIDE (B). The cutting efficiency were indicated top left.
(TIF)

S1 Table List of primers used for RT-qPCR. (PDF)

S2 Table List of primers used for ChIP-qPCR. (PDF)
