# Supplementary figures and images for: EBNA2 driven enhancer switching at the CIITA-DEXI locus suppresses HLA class II gene expression during EBV infection of B-lymphocytes
Source: PLoS Pathog. 2021 Aug 5;17(8):e1009834. doi: 10.1371/journal.ppat.1009834 (PMC8370649; doi:10.1371/journal.ppat.1009834)

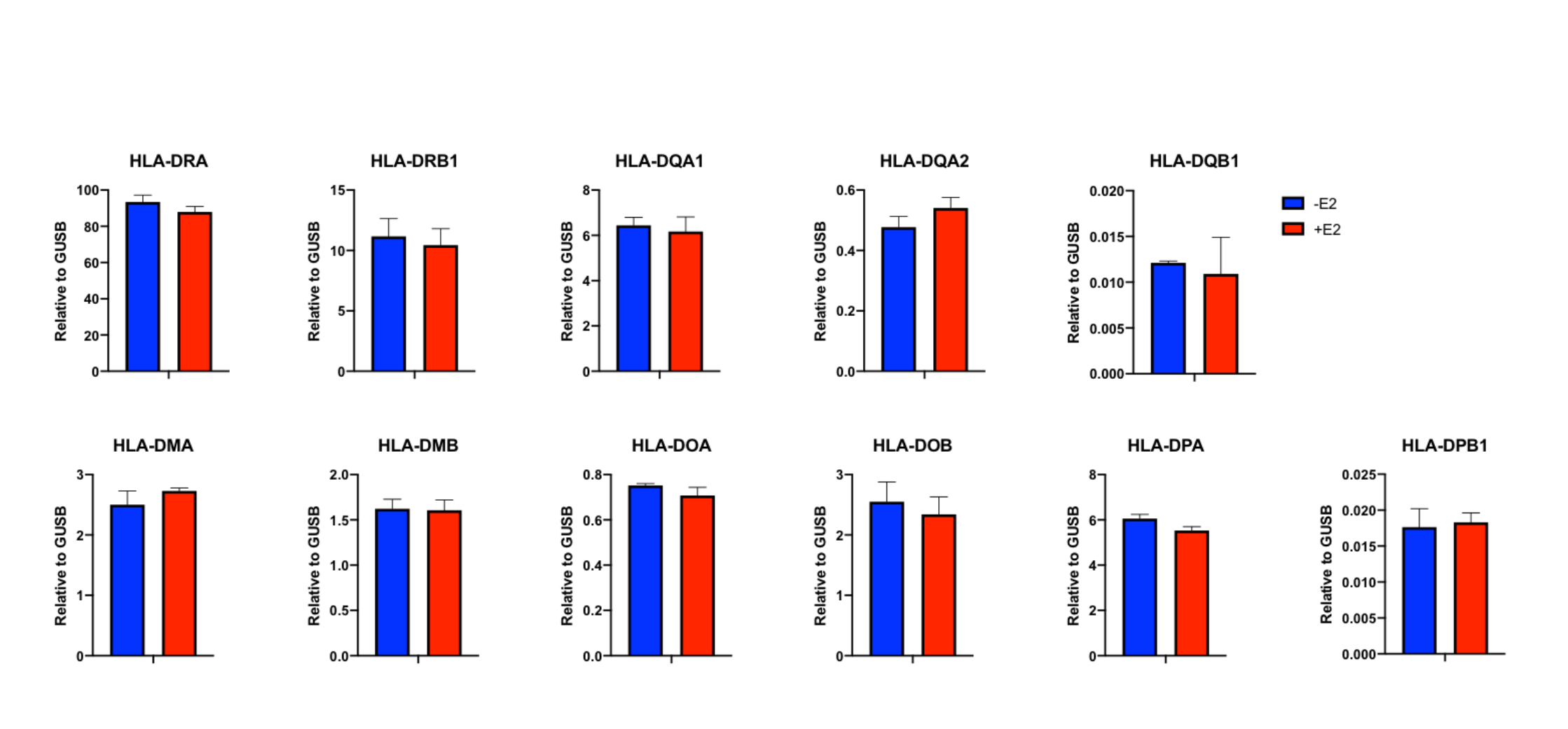

Supplement: S1 Fig — To rule out the potential impact of estradiol on HLA transcription, LCL352 was treated with (+) or without (-) estradiol for 48 hrs and then assayed by RT-qPCR for HLA-II gene transcription. (TIF) [file ppat.1009834.s001.tif]

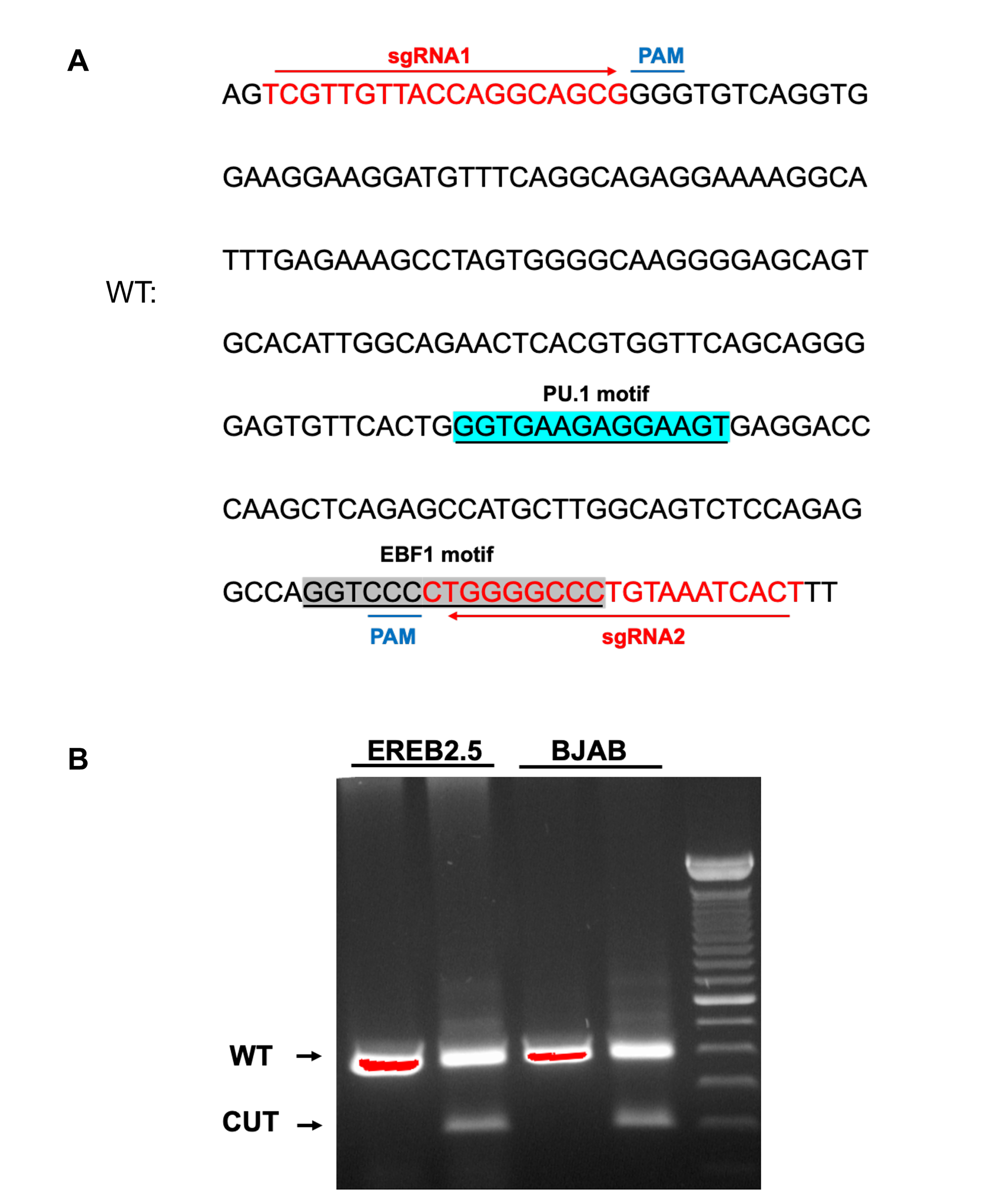

Supplement: S2 Fig — (A) Schematic diagram showing gRNA positions and location of EBF1 and PU.1 motifs. (B) After CRISPR deletion of EBNA2_BS, genomic DNA was PCR amplified and verified for deletion efficiency. (TIF) [file ppat.1009834.s002.tif]

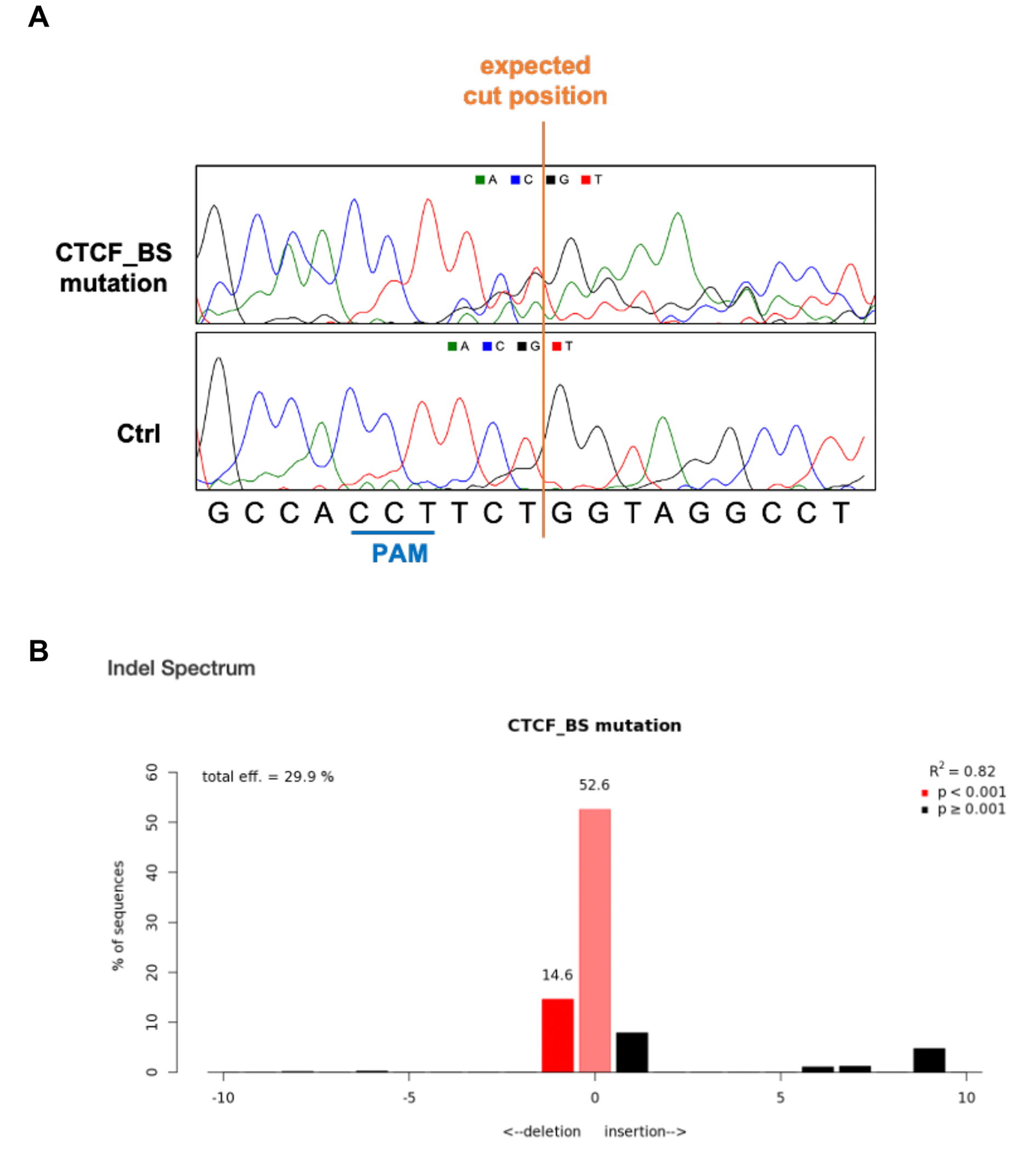

Supplement: S3 Fig — To confirm the CTCF-BS mutation, PCR was performed to amplify the gRNA targeted region. The purified PCR fragments were Sanger sequenced (A) and analyzed using TIDE (B). The cutting efficiency were indicated top left. (TIF) [file ppat.1009834.s003.tif]
