# Supplementary material for: EBNA2 driven enhancer switching at the CIITA-DEXI locus suppresses HLA class II gene expression during EBV infection of B-lymphocytes
Source: PLoS Pathog. 2021 Aug 5;17(8):e1009834. doi: 10.1371/journal.ppat.1009834 (PMC8370649; doi:10.1371/journal.ppat.1009834)
Supplement: S1 Table — (PDF) [file ppat.1009834.s004.pdf]

RT-qPCR primers	
HLA-DRA_F	ATGGCCATAAGTGGAGTCCC
HLA-DRA_R	TCGCCTGATTGGTCAGGATTC
HLA-DRB1_F	CCGGGCTGTTCATCTACTTC
HLA-DRB1_R	CTGGGGCAGAAAGTTCTTCTT
HLA-DPA1_F	GGGGACCCTGTGAAATACTGT
HLA-DPA1_R	CAGCTGGAGTTCAGATCTCTCC
HLA-DPB1_F	ACAGGATGTGCAGACACAACT
HLA-DPB1_R	CCTTCTTGGAGGGGGAAACAT
HLA-DMA_F	GTGGCAAGAAGGTATGGGTCA
HLA-DMA_R	TGGCCACATTGGAGTAGGAG
HLA-DMB_F	TGGGGATCACTGACCAACAG
HLA-DMB_R	TCACAGGCTCCCTCGTGTTA
HLA-DOA_F	GGGTTCCACACCCTGATGAC
HLA-DOA_R	AGACTGGTAGAAGGCGGGTC
HLA-DOB_F	TCTGACCCGACTGGATTCCT
HLA-DOB_R	TGCACCTTTTCTGTCCCGTT
HLA-DQA1_F	CGCAGGGTGCACTGAGAAA
HLA-DQA1_R	GCGTTTAATCATGATGTTCAAGTTG
HLA-DQA2_F	GTGGGAAAACACACCTTGGAA
HLA-DQA2_R	CATTGGTGGCAGCGGTAGA
HLA-DQB1_F	GCGCGTGCGTCTTGTG
HLA-DQB1_R	TCGAAGCGCGCGTACTC
HES1_F	GAAGCACCTCCGGAACCT
HES1_R	GTCACCTCGTTCATGCACTC
c-Myc_F	TTCGGGTAGTGGAAAACCAG
c-Myc_R	CAGCAGCTCGAATTTCTTCC
CIITA_F	CTGTGTCACCCGTTTCAGG
CIITA_R	GAAAGGCTCGATGGTGAACT
DEXI_F	ATCTGGACGGGGATGGTT

DEXI_R	TTTGAGGGGAGCAGGTAGG
GUSB_F	CGCCCTGCCTATCTGTATTC
GUSB_R	TCCCCACAGGGAGTGTGTAG
