# Supplementary material for: EBNA2 driven enhancer switching at the CIITA-DEXI locus suppresses HLA class II gene expression during EBV infection of B-lymphocytes
Source: PLoS Pathog. 2021 Aug 5;17(8):e1009834. doi: 10.1371/journal.ppat.1009834 (PMC8370649; doi:10.1371/journal.ppat.1009834)
Supplement: S2 Table — (PDF) [file ppat.1009834.s005.pdf]

ChIP-qPCR primers

CIITA-pIII_F	TCACCAAATTCAGTCCACAGTAAGG
CIITA-pIII_R	GCCCCAAGCGGTCAGATTTC
DEXI-promoter_F	CGAATAGGGGGAGGTAAAAGAG
DEXI-promoter_R	GGAGAAACGGAGTCCCAAAG
EBNA2, EBF1&PU.1_BS_F	TGGGTGAAGAGGAAGTGAGG
EBNA2, EBF1&PU.1_BS_R	TCAGTGGCTCCCTACTGTTC
CTCF_BS_F	AAATATTGCTCCTTTTCCCCC
CTCF_BS_R	AGTCCGCTCATTACCTCTG

ChIP primers - enhancers at HLA-II loci

p1_F	TCCCTTACGCAAACTCTCC
p1_R	GACTCTTTAACCCCAATCTGAC
p2_F	GCAGCATCACTTGTCTCC
p2_R	CATCGTCAGCCATTCTTCTC
p3_F	AACCACATTAACATGAAACCTTGA
p3_R	CTGCCAGAGACAAATGATGC
p4_F	CTCCATGGGCCTCCATTGTT
p4_R	GGTCTACCCCGTGAGATTGC
p5_F	ATTTTCCCTCCATCCCCCTC
p5_R	TGTCTCCTTGCTCACCTTC

ChIP primers - enhancers at CIITA loci

p1_F	AACAGCCAGTGAAATGCAG
p1_R	TACAGACCCATAGCAAGCC
p2_F	TGCTCTTTTCCTGCTTTCTC
p2_R	CTGTCCTCTTTCTTCCCCTC
p3_F	ATTTCTACCCCACTCCTCAC
p3_R	CCTTGGCCTCTTTACTTCCTC
p4_F	ATCTCACCCCCAACCATCTC

p4_R	GCAGGAACCCAAACATAACC
p5_F	ACAAATGCACCTGTTGCC
p5_R	GATTTTTCTACTACCCCCTCAC
p6_F	GAAGAGGAACAAGGAGTGAAG
p6_R	TTGGGAGAATGGAGGATGAG
p7_F	TGTGGCATTAGGAAGCAGG
p7_R	TGAAGGTGACACAGCAAGG
p8_F	ATGGGATTAGACAGAGTGAGG
p8_R	TGATTAGCAGGTGGGCAAG

CRISPR screen PCR primers

CTCF_BS KO_F	AAATATTGCTCCTTTTCCCCC
CTCF_BS KO_R	AGTCCGCTCATTACCTCTG
EBNA2_BS KO_F	CCACTATGTCACGTCTAAGTTC
EBNA2_BS KO_R	TCAGTGGCTCCCTACTGTTC
